# The Cetacean Sanctuary: A Sea of Unknowns

**DOI:** 10.3390/ani14020335

**Published:** 2024-01-21

**Authors:** Jason N. Bruck

**Affiliations:** Department of Biology, Stephen F. Austin University, SFA Station, Nacogdoches, TX 75962, USA; jason.bruck@sfasu.edu

**Keywords:** cetacean, sanctuary, whale, welfare, sea pen, naturalistic enclosures

## Abstract

**Simple Summary:**

Sanctuary is a term with implicit meaning associated with refuge and safety. In the animal husbandry setting, we have used this term to describe many different types of enclosures, with very little standardization of what this term should mean in terms of physical space, much less welfare in a broad sense. Here, I consider how extreme space, the deemphasis of the human–cetacean relationship, breeding restrictions, donation-based funding models, and current practices in public messaging create potential challenges for cetacean sanctuaries moving forward. I then offer experimental approaches to assessing the potential effectiveness of cetacean sanctuaries in improving cetacean welfare. To ensure the success of sanctuaries in maximizing animal welfare, it is essential to critically evaluate sanctuary standards against what is currently known about animals under managed care, as well as to determine what data are necessary to fully evaluate animal welfare in sanctuaries before these facilities are constructed.

**Abstract:**

Housing cetaceans in netted sea pens is not new and is common for many accredited managed-care facilities. Hence, the distinction between sanctuary and sea pen is more about the philosophies of those who run these sanctuary facilities, the effects of these philosophies on the animals’ welfare, and how proponents of these sanctuaries fund the care of these animals. Here, I consider what plans exist for cetacean sanctuaries and discuss the caveats and challenges associated with this form of activist-managed captivity. One goal for stakeholders should be to disregard the emotional connotations of the word “sanctuary” and explore these proposals objectively with the best interest of the animals in mind. Another focus should be related to gauging the public’s understanding of proposed welfare benefits to determine if long-term supporters of donation-based sanctuary models will likely see their expectations met as NGOs and their government partners consider moving forward with cetacean sanctuary experiments.

## 1. Introduction

The public’s changing attitudes on the treatment of animals have necessitated a reevaluation of the relationship humans have with the species under our care. With that has come changes in how we assess and implement animal welfare. In this rapid reevaluation, there has been tension between philosophical ideals and practical realities, especially as public expectations have induced change under truncated timeframes. Some changes have been for the positive, like the widespread use of enrichment in zoos and aquaria, which has correlated to improved animal lifespans [1]. Some changes, even those made with the best intentions, have had net negative effects on animal wellbeing, as an incomplete understanding of the biology and behavior of the animals we mean to help have led to interventions that were less successful than hoped. Such is the case with attempting to relocate modern egg-laying chickens from battery cages to free-range environments, as the breeding for increased egg production [2] has made these osteoporotic animals unsuited for uncaged housing due to a lack of skeletal rigidity [3]. Therefore, the public outcry against factory farmed egg production has to be considered alongside the reality that moving chickens from battery cages to free-range enclosures necessitates gene modification techniques to see the increased welfare benefits associated with cage-free housing [4]. This example highlights the importance of rigorously collected data to anticipate the complications of potential welfare interventions and to consider how animal care is benefited or compromised by a given change, as well as whether long-term benefits can arise from a new welfare system. There is a need to consider the available science when contemplating animal management changes for philosophical reasons to maximize the odds that welfare improves, especially when the pressure of society demands quick action, making performing welfare due diligence even harder.

Cetacean welfare in zoos and aquariums has been under public scrutiny for some time; however, over the last 10 years, with the rise of social media and the production of two influential documentaries: The Cove and Blackfish, members of the public have begun to more earnestly question the ethics of cetaceans in professional care [5,6]. This is despite the improvement in care seen over the last few decades preceding the release of the aforementioned documentaries [5,7,8,9]. Increased public scrutiny has led to a greater will for the public to entertain the notion of seaside sanctuaries as something of a compromise between naturalistic full-release (see Keiko [10]) and a pool enclosure. A cetacean sanctuary has been recently defined by the Global Federation of Animal Sanctuaries (GFAS) as a net/sea pen style habitat in naturalistic conditions [11]. It is true that facilities exist all over the world with net pen-like housing. What is said to distinguish a cetacean “sanctuary” from a sea pen by GFAS standards is a set of ethics that includes a strict policy of no-breeding, a deemphasis on the public’s exposure to the animals, expansive enclosures, a reduced focus on financial gain, and in some cases, a reduction in training/human–animal interaction (see Section 1.2 *Standards of a Sanctuary* for expansive discussion) [5,6,11,12]. These distinctions are important because they will have welfare consequences moving forward. As such, this is not principally a discussion about whether cetaceans should be housed in naturalistic sea pens vs. pools/tanks (see Section 2 for distinctions of housing types). This is a discussion about whether the naturalistic housing defined as a sanctuary by the GFAS standards is likely to offer better, worse, or net neutral welfare conditions relative to the naturalistic housing in sea pens common today in facilities all over the world. The maintenance of animals in naturalistic sea pens or lagoons already presents challenges, with weather and the elements as the foremost concerns [13]; however, the prescribes of the sanctuary ethos have the potential to exacerbate existing challenges in ways that may lead to less naturalistic husbandry and, ultimately, poorer animal wellbeing if not considered presently.

### 1.1. Concrete Pools, Naturalistic Lagoons, and Netted Sea Pens/Seaside Sanctuaries: What Are the Differences?

**Figure 1:**
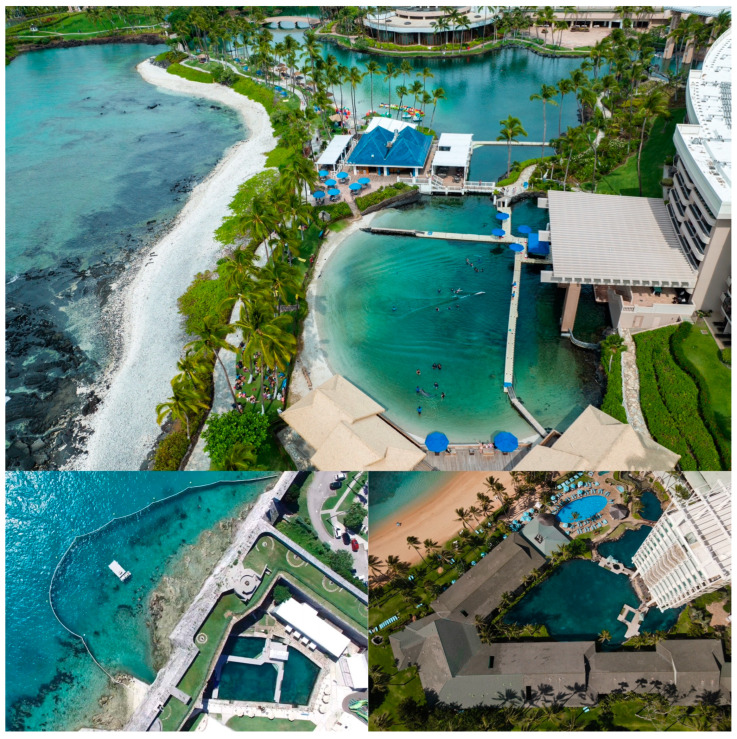
Three naturally fed lagoon facilities. (**Top**) Dolphin Quest Hawaii located on the Big Island of Hawaii at the Hilton Waikoloa. (**Bottom left**) Dolphin Quest Bermuda located in the National Museum of Bermuda. (**Bottom right**) Dolphin Quest Oahu located at the Kahala Resort in Oahu. Photo Credits: Dolphin Quest.

According to data compiled from Cetabase.org, net pens account for 15% of managed cetacean habitats globally, with another 4% categorized as lagoon facilities [14]. I have alluded to the differences that proponents of seaside sanctuaries envision as important in defining a sanctuary vs. a sea pen. However, in considering the properties of the physical habitat, there is really no difference between these enclosure types in terms of animal environmental exposure. Both are defined as netted areas of ocean habitats for the purpose of enclosing cetaceans. What offers more contrast is the comparison among pools, lagoons, and sea pens (GFAS sanctuaries or not). While I will mostly focus this paper on the distinctions between sea pens and GFAS sanctuaries, it is important to understand how these animals are currently housed under professional care to understand what a transition to a GFAS sanctuary might look like for some of these animals. A pool enclosure is typically constructed of concrete and inland of the ocean (although in some cases not too far from the ocean). Sea pens/GFAS sanctuaries are necessarily coastal and are fed by ocean water where the net is placed. Lagoons represent something of a mix of these two types. These are facilities fed by natural waters, but the enclosure is not a net. This can include habitats that are human made, either as structures or as bodies of water separated somehow from the wider ocean (Figure 1).

**Figure 2:**
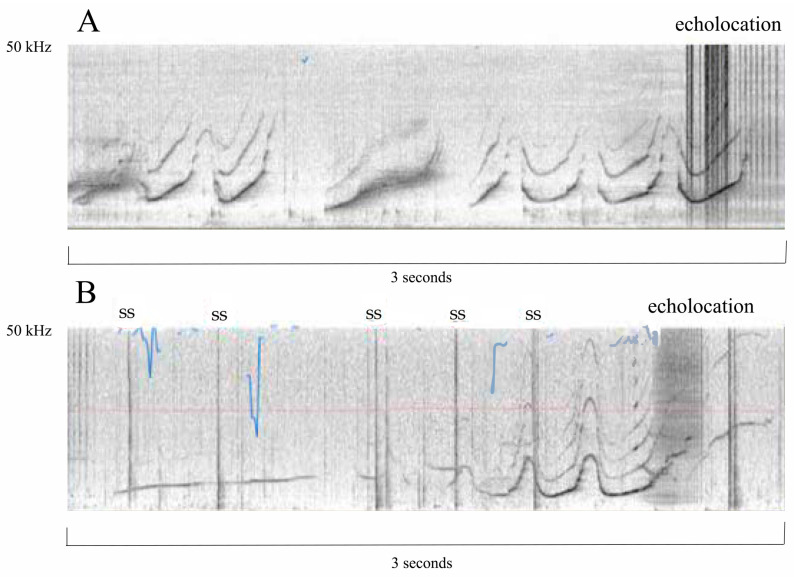
Analogous contours taken from both a concrete pool faculty (**A**) and a lagoon facility (**B**). Samples were randomly selected from the 53rd recording (collected in 5-min sections) taken at each facility. Not only do the dolphins each express similar contours, but animals at both facilities echolocate regularly (demonstrated at the end of both spectrograms in virtually the same place). Only the snapping shrimp (marked by SS) reveals which facility is a lagoon. Frequency (0–50 kHz) appears along the *y*-axis, and time (3 s) appears along the *x*-axis. Recordings taken and spectrograms prepared in PRAAT [19] by Jason Bruck.

Pools differ from lagoons and sea pens/sanctuaries in that they require filtration, which can be noisy [15]. However, they are typically protected from many environmental hazards that are unavoidable with open exposure to the ocean (red tides, hurricanes, oil spills, anthropogenic ship noise, etc.). One criticism of concrete pools is that there are thought to be negative effects related to cetacean communication and/or echolocation with sound refraction [16]; however, no study I am aware of has shown this in zoological facilities, and there are publications [17,18], as well as anecdotal evidence, that demonstrate that cetaceans living in pools echolocate without difficulty (Figure 2).

### 1.2. The Standards of a Sanctuary

On 28 June 2023, the GFAS released standards for cetacean sanctuaries version 1 [11]. Borrowing heavily from a 2013 GFAS standard for marine mammal sanctuaries [20], The GFAS standards for cetacean sanctuaries aim to provide a comprehensive set of guidelines for the ethical treatment of whales and dolphins in sanctuary settings. These standards are meant to apply to animals currently under human care (belugas, orcas, and multiple other dolphin species) more than great whales, beaked whales, or deep-diving whales, which are excluded due to their unique needs. The authors emphasize that standards should prioritize replicating natural habitats. The standards encompass areas such as habitat design, social dynamics, veterinary care, and education. Proponents of these new standards argue that by emphasizing a form of naturalism, their standards aim to ensure the highest level of care and promote the long-term welfare and survival of these marine mammals in sanctuary environments. Proponents of the sanctuary model espoused in the GFAS standards argue that cetacean sanctuaries and the current methods of housing differ primarily in their purpose, approach to animal care, and ethical considerations.

A. Purpose and Conservation:

The authors of the GFAS standards argue that cetacean sanctuaries are designed to meet the animals’ physical, psychological, and social needs more closely by offering larger, more naturalistic habitats. The emphasis is on minimizing stress and promoting wellbeing. Zoos, historically criticized for confining animals in what some perceive as limited spaces, have evolved over time to focus on better enclosures and enrichment, but concerns remain among some regarding the ethical implications of zoo-based professional-care [5]. Sanctuaries aim to rehabilitate and/or provide a lifelong home for animals that cannot be released into the wild due to injuries, history under professional care, or other factors. It is argued that sanctuaries would place an emphasis on conservation and welfare that would take precedence over entertainment. Zoos often prioritize public entertainment and education alongside conservation efforts.

B. Interaction with the Public/Trainers:

Cetacean sanctuaries generally limit or avoid public interactions, aiming (according to the GFAS standards) to reduce stress on the animals and maintain a more natural environment (both physically and socially). For sanctuaries, a deemphasis on non-conspecific interactions may be desired, as ideal natural socialization is typically seen as whale-to-whale rather than whale-to-human. In contrast, zoos often offer public viewing and interactions as part of their educational and revenue-generating activities.

C. Ethics Around Breeding:

Cetacean sanctuaries, as defined by the GFAS standards [11], would not allow for the breeding of animals. This may involve sex segregation or long-term use of birth control [21].

D. Funding:

While it is not an ethos of the GFAS standards, funding models that rely less on public interaction necessitate greater donation revenue. Each of the sanctuaries or proposed sanctuaries under the GFAS authorship [11] relies on fundraising for part or almost all their operating expenses [22]. 

I will start off by suggesting that any form of housing that improves the quality of life for animals under human care should be considered seriously. The critiques I offer of the GFAS standards do not preclude the possibility of successfully housing animals in ways similar to the means described by the GFAS. However, the GFAS standards as they currently exist are missing critical elements necessary to promote success in this new form of housing whales. I will preview these concerns here with a deep dive into each element of what separates a GFAS sanctuary from a currently existing sea pen throughout the remainder of the paper, discussing concerns and areas to consider in each element. 

As a document, the GFAS cetacean standards offers prescriptions but very little justification for those edicts. For example, the GFAS guidelines indicate that *the sanctuary’s area should be equivalent to 4047 m^2^ (1 acre) per 16 km (10 miles) of average daily swim distance in the wild for the species being housed (note: this is a minimum and additional space is encouraged)* [11], (p. 8). However, there are no references highlighting how this figure was derived (the same is true for depth determinations). Contrast this with the Association of Zoos and Aquariums (AZA) standards for habitat space requirements for elephants, in which the standard is given, then a means of measuring the success of following the standard, followed by a rationale/explanation for the standard that includes best science and citations [23], (p. 46). The AZA approach is more comprehensive and better justified. Part of the reason for this is likely that zoos predate the AZA. This means that standards were developed based on data and observation of an already existing enterprise. The GFAS standards, however, predate any successful cetacean sanctuary facility. This then begs the question: how do we know the edicts of the GFAS cetacean standards qualify as best welfare practices? In her 2023 white paper, Lori Marino, President and Founder of the Whale Sanctuary Project, attempts to rebut criticisms toward the sanctuary model outlined in the GFAS standards [24]. In responding to criticisms of the lack of sanctuary standards (which did not exist specifically for cetaceans until the 2023 publication of the GFAS standards, making this criticism valid up until the summer of 2023), she said that

*“This document [the GFAS standards] is based on more relevant and, in many ways, stricter criteria for well-being than currently exist in the standards for the marine park industry that have been established by the Association of Zoos and Aquariums”.*

Unfortunately, the white paper does not address how the GFAS standards are better than the AZA standards in terms of actual welfare data, nor does she clarify how the GFAS standards are more relevant, as they do not include research or citations to justify welfare standards. For example, one cannot simply assume that more space requirements necessarily indicate better welfare. Stricter criteria implies that the standards are connected to demonstrated welfare outcomes. There is no evidence for this in the GFAS standards; however, there are areas of concern and opportunities for data collection that may justify the standards outlined by the GFAS.

### 1.3. The Five Domains of Animal Welfare

The five domains of animal welfare include housing, nutrition, veterinary care/health, behavior/social/training, and mental state/experiences [25]. Some of these domains are relevant to a discussion of sea pens vs. sanctuaries, and others are less so. In terms of nutrition, it can be assumed that animals in a sanctuary will receive the same restaurant-quality fish afforded to non-sanctuary animals. Hence, I will not address the nutrition domain here. Other matters, such as behavior/social/training, housing, veterinary care, and mental/state experiences, are inextricably linked to issues surrounding the massive habitat size of a sanctuary and the sanctuary’s viewpoint on natural interactions vs. human animal interactions. Therefore, where appropriate, matters of the five domains that are connected to specific prescriptions of a GFAS sanctuary (vs. existing sea pens) will be considered jointly. Specific matters of health and behavior related to the effects of breeding bans in GFAS sanctuaries will be considered together, as those issues are confined to a narrow set of prescriptions (birth control or sex segregated housing). Finally, my discussion of funding and sanctuary messaging have more downstream effects on welfare; therefore, it is likely inappropriate to directly connect those elements with these five domains explicitly. This paper is, therefore, arranged using the criteria that separate a GFAS sanctuary from a currently existing sea pen or lagoon facility. 

## 2. Space and Human Interactions: Implications for Housing, Health/Veterinary Care, Behavior, and Mental State

At first glance, one might consider maximizing space to be an ideal scenario for providing cetaceans naturalistic living conditions. The ocean is large, and therefore, one might assume that cetaceans should want to inhabit large enclosures. However, this logic does not take into consideration the fact that most cetaceans under managed care have no experience with the open ocean, or at least no recent experience in the case of long-term managed animals (in North America and Europe, healthy animals have not been taken from the wild in decades). What, then, does this mean for the whales’ expectation of a habitat, and how should we expect rearing condition (under managed care vs. the wild) to affect behavior and animal wellbeing? Would instinct drive some innate desire to inhabit large, ocean-sized spaces, or does life-long experience in a pool lead to the development of dolphins with a learned preference for smaller enclosures that facilitate closer proximity to human caregivers? While no proponents of the GFAS cetacean sanctuary standards advocate the release of long-term captive or captive-born individuals, some previous release attempts may be instructive in anticipating how animals from pools, lagoons, or current sea pen environments may react to expansive sanctuaries, especially as they relate to human dependence post-move to these new environments. We know that short-term rehabilitations of young animals can lead to some degree of reintegration, as a <1 year old male bottlenose dolphin from the Indian River Lagoon in Florida was released after three months of rehabilitation under professional care; although, the ultimate success of the project was undeterminable after contact was lost with the animal after seven days [26]. A more focused version of this scenario occurred as an experiment in the late 1980s and early 1990s called the “Welcome Home” project, in which researchers took two young bottlenose dolphins and brought them under professional care for two years and then released them back to the wild. For the first month, the two dolphins interacted with each other almost exclusively; however, in the second month after release, the animals reintegrated to their native social groups, spending less time with each other [27]. Given that these were young animals who were carefully chosen for this experiment with known social histories in a well-studied population, their capture and release was set up for success in a way that other projects would likely struggle to emulate. This brings us to our final story of release, which took place off the coast of Perth, Australia, in the early 1990s. Led by then Ph.D. student, Kelly Waples, and Dr. Nick Gales, the goal was to rehabilitate and release nine bottlenose dolphins (three adult males, three adult females, and three zoo-born female juveniles) from a marine park that was closing. Here, the demographics of the animals were different than those of the previous release scenarios, which featured long-term captive animals, as well as dependent calves. The release was attempted in stages, with a sea pen stage as part of the plan (inspired by the Welcome Home project). While some of the older animals showed some proficiency in catching fish in the pool, that same success was not seen in the sea pen. Furthermore, when the animals were released in January 1992, most of the animals showed weight loss, failure to thrive, significant begging behaviors toward humans, and even the loss of a calf born during the sea pen stage. Some of the animals were recovered and brought back to the sea pen, while others were lost and never seen again [28]. Dr. Waples would later write an article on this experience for PBS Frontline [29], where she stated:

*“My involvement in this project also changed my attitude and perception of releasing captive animals into the wild. Initially I strongly favored release, believing that to return animals to their natural habitat after years of confinement was an admirable goal as it seemed to guarantee improvement in living situation and welfare. But as the release developed and I was faced with the difficulties encountered by the dolphins and their obvious decline in welfare, my attitude changed. Was release really in the dolphins’ best interests? We talk about animal welfare and animal well being and (although there are a variety of definitions), mean that animals should be dealt with humanely, that they must not be made to suffer or die unnecessarily and, that any causes of suffering must be minimized. But the entire release process is bound to be full of intense stresses and potential risk of mortality, higher than the risks incurred in captivity”.*

Importantly, the Perth release was one of the earliest pieces of evidence available that showed that humans are a very hard thing for cetaceans under long-term professional care to quit. The frequent begging and human associations seen in these releases speak to the importance of learning history, as well as the inability of natural trappings to erase many cetaceans’ dependances on human socialization and interaction. This would be a pattern we would see again, with implications for what we should expect of cetaceans placed in multiacre sea pen sanctuaries. 

The strong role of experience in driving behavior and cognition is evidenced by cetacean acoustic development and social transmission of behavior, meaning that cetaceans are very much a product of their learning environment [30,31,32,33,34,35,36]. Animals born under professional care know nothing of the ocean, and it stands to reason that not all cetaceans will react well to large enclosures, even in the context of being placed in a sea pen. Because space and human contact are inextricably linked (theoretically more space could mean less human contact), and critical considerations of both in the setting of a sanctuary challenge the narrative that cetaceans born into pools will uniformly display more wild-like behaviors in a sea pen simply because they are placed into the latter, we will consider these two factors together.

We have seen the push and pull of learning history vs. genetics recently with two belugas (*Delphinapterus leucas* [37]) placed under the care of the Sea Life Trust, a charitable arm of Merlin Entertainment (Table 1). The whales, named Little Grey and Little White, were captured as young animals from the Arctic coast of Russia and taken to a coastal research facility, first, and then moved to a facility in China. When Merlin Entertainment purchased Changfend Ocean World in Shanghai in 2012, the company made clear their intentions to move the animals to what was termed a “more natural environment” by Andy Bool, the head of the Sea Life Trust [38]. In June 2019, the whales were taken first by truck, then plane, then truck again, and finally, by ferry from China to the Beluga Whale Sanctuary in Heimaey Island, Iceland. Upon arrival in Iceland, the whales were placed in a quarantine care pool for what was supposed to be period of monitoring and resting after their transit. It would not be until 8 August 2020 (~14 mo. later) that the whales would be placed in the actual sea pen enclosure in Klettsvik Bay, Iceland. The full year spent in the transition pools was attributed to “their progress and the changing weather conditions” [39]. On 8 December 2020, the animals were moved back into the transitionary pools with weather cited as the reason [40]. It should be noted that the mean temperature in Iceland is about 9 °C in the summer and −3 °C in the winter, with frequent storms and rain characterized by an annual precipitation ranging from 400 to 4000 mm on average annually, depending on location [41]. While Sea Life Trust noted that the animals were progressing well with their adoption of the new environment, “Little Grey and Little White have surprised us with some of the positive ways they have adapted to their new natural environment (catching live fish, exfoliating on the seabed, interacting with other wild animals)”, no details were given as to exactly how the animals were adapting [40]. ITV.com noted that there was a slowness in how Little White acclimated to the bay relative to Little Grey. This meant that an intermediate habitat in the sea pen would need to be created, as it was felt that Little White was not able to immediately adjust to the added space that was assumed to be preferred. Audrey Padgett, the then General Manager of the Sea Life Trust Beluga Whale Sanctuary, was quoted by ITV.com saying “It gives her and other future belugas a middle step in getting used to a controlled indoor environment and then used to all the changes in an outdoor one such as daylight and changing tides” [42]. This was the first inclination that the whales would not necessarily welcome the added space of a sea pen by default and that variation in response to space might be individually distinct amongst different cetaceans. In that same ITV.com article, it was mentioned that it would not be until spring 2022 that the whales would potentially have another chance to go to the sea pen due to delays in obtaining materials for the inner net system meant to transition Little White [42]. However, on 24 August 2022, a press release from the Sea Life Trust noted that just hours before the whales were due to move, the Beluga Whale Sanctuary team were surprised to discover that a contractor’s boat had sunk in the bay in the early hours of Sunday, 14 August, leaking oil and fuel into the sanctuary [43]. Thus, it was not until the middle/end of May 2023 that the animals were moved outside. These delays highlight the difficulties associated with housing cetaceans in sea pens, and while difficulties like this can occur at existing sea pen facilities, some of the problems sanctuaries may face are exacerbated by choosing remote locations to keep people away from the animals and by using thousands of feet of netting that must be maintained in often difficult environments. When the animals were eventually transported to the sanctuary, Little Grey almost immediately showed signs of appetite loss, as announced by a 25 May Facebook social media post from the Head of Sanctuaries, Jana Sirova. On 3 June 2023, an update on the animals was made by curator Courtney Burdick announcing that both whales were again in their temporary pool. This time it was clear that there were physiological effects resulting from the move; Little Grey was discovered to be harboring ulcers [44] (a condition that would persist until December 2023). Upon discussing the matter with trainers who were on site, it was clear that the animals’ behaviors were breaking down, as they were no longer responsive to animal care personnel. The whales would approach the professionals for only as much time as it took to throw a fish in their mouth before swimming away [45]. 

In many ways, the journey and ordeals of these two whales can be framed as something of an experiment; however, in any experiment, one must be open to either accept or reject a null hypothesis. In the case of cetacean sanctuaries, we are left with a difficult question: how do we know when we have failed to support the hypothesis that the sanctuary model is a universal best approach to animal welfare? In this case, just from the publicly available comments and press releases, both whales, at different times, seemed to have had adverse reactions to the large sea pen environment (although other potentially confounding factors may exist but are unknown to this author). This seemingly impacted these animals’ ability to interact with their animal care team, which likely exacerbated health issues, at least for Little Grey. This brings doubt to the narrative that just maximizing space should be the primary consideration when considering cetacean welfare. Limited as it is, when we consider the research on cetacean welfare in which space is a consideration, cetaceans under professional care do not seem to prioritize habitat size, focusing more on interactions with their human caregivers [7,46]. A deemphasis on these relationships in favor of a less preferred aspect of their environment seems antithetical to proper animal care in professionally managed cetaceans. It is naive to assume that cetaceans born under managed care will forgo all their learned history and adopt more wild-like behaviors and preferences upon placement in a sea sanctuary (see Section 7 for recommendations on habitat size and type). 

Regarding the cetacean–human bond/dependence, there is evidence to suggest that there is a stubborn permanence to the effect of human contact on cetacean behavior that persists despite placement in or a continued existence in the naturalistic conditions of a sea pen or the ocean at large. Wild cetaceans can quickly acclimatize to human feeding, with often deadly results [47,48]. These “begging” behaviors are often learned quickly, can be pervasive, and spread through populations rapidly [49,50] despite the dangers of boat motors, vessel strikes, and willful human abuse [47]. Therefore, it seems, cetaceans accustomed to human interaction remain accustomed, even when it is to their long-term detriment. This has implications for how we should interpret the GFAS standards. Is a deemphasis of the human–animal bond truly what is in the best interests of these animals (implicit or assumed as the animals are supposed to take on more naturalistic behaviors)? With more space and fewer humans, will we have whales that, in behavior and manner, more embody their wild counterparts? Or are we forgetting these animals’ learned histories and setting them up for worse welfare outcomes?

**Figure 3:**
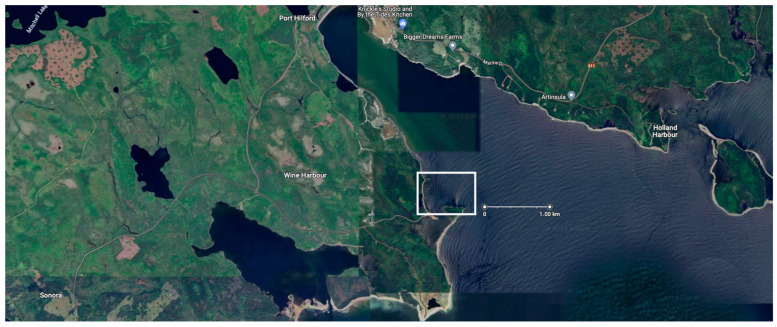
Proposed location for the Whale Sanctuary Project in Port Hilford, NS, Canada (white box), with 1 km shown for reference. Image obtained from Google Maps, imagery ©2024 Airbus CNES/Airbus, Landsat/Copernicus. Maxar Technologies. Man data ©2024 [58]. WSP website shows that the area of this enclosure will be 484,000 sq yards (~100 acres/404,685 sq meters) [59].

Moving whales to a sanctuary is a step away from a pampered existence and a step toward elements of the wild characterized by uncertainty of food, loss of environmental safety, increased risks of predation/disease, and competition for resources (factors for wild whales that are known to cause morbidity and mortality, as well as distress [51,52]). How big that step is depends on many factors, including size of the environment, weather, knowledge and ability of the care staff, and most importantly, the guiding philosophy of the sanctuary itself. If it is the philosophy of the sanctuary managers that the whales’ relationships with humans are to be deemphasized in the hopes of making room for the whales to bond with local flora and fauna (perhaps thinking the animals may benefit from a genetic propensity to embrace naturalistic conditions), then that step is a significantly bigger one. There is a caveat to space and relationships that deserves mention, and that is the concern about accessing sick animals. This issue can be exacerbated if training and human–animal bonding are not emphasized, as a sanctuary facility with acres of space may be confronted with animals that are no longer active participants in their own health care and are unable or unwilling to be brought to medical areas (Figure 3). These issues are abated in facilities like the U.S. Navy Marine Mammal Program, as those animals are kept in more manageable spaces (30 ft × 30 ft, with daily open ocean work), are perpetually trained (including transport training), and have quick access to top medical equipment, including diagnostic imaging like CT [53,54,55]. Again, the magnitude of this concern fluctuates with the level of independence expected of sanctuary animals and the literal size of the space from which the animals must be accessed. This could be more problematic in the case of sanctuary housing pool-born animals, who may not present with the same degree of disease resistance as animals born in the ocean [52]. One could argue that these risks are contrasted with the animals’ increased opportunities for behavioral choices and the value of having the animals in natural conditions. However, what is natural to a pool-born cetacean, and is our conception of “cetacean natural” good for their welfare? What if a pool-born whale moved to a sanctuary prefers to interact with humans and would choose that behavior if offered? Does the sanctuary really offer more behavioral choice if that is taken away? What are the welfare consequences of this? Dr. Heather Browning makes the point that we should not necessarily assume natural behaviors are the best indicator of good welfare in managed settings [56]. In this case, this might take the form of using basketballs for dolphins to bounce around in a pool rather than attempting to use something a dolphin may encounter if it were in the ocean (live fish or seaweed perhaps) because the dolphin prefers the ball. What is good enrichment for an animal under managed care does not necessarily have to be what is natural, as the concept of what we think a dolphin should think is natural might elude an animal born in the company of humans. I interpret her work as a caution against a naturism fallacy when it comes to considering what is good for animals (i.e., the idea that what is found in nature is good by virtue of it being natural and wild). Given the propensity of formerly captive animals to still choose to embrace relationships with humans when they have a literal ocean of choices of other things to occupy themselves with, we must take this consideration seriously when determining what is preferred (i.e., what would they really choose if they had their way) by a long-term captive animal or a pool-born whale. As a case study, Keiko the killer whale (*Orcinus orca* [57]) provides a powerful example of the strength of the human–cetacean bond, once formed, and how difficult it is to extinguish [10]. His relationship with humans was ultimately the *Achilles heel* in his release plans and provides some instruction now in guiding how the human–animal bond should be considered in sanctuaries.

Keiko’s story was unique. In 1996, he was an Icelandic orca living in Mexico City, Mexico, about to begin a journey that would captivate the world and, ultimately, cost him his life [10,13]. He rose to prominence as the star of the Warner Bros. film *Free Willy*, a story of an orca under managed care who is successfully released into the wild by a boy who befriends the whale. There was a push from the public to see life imitate art, and in January 1996, Keiko began what would be a multi-trip odyssey, first taking him from Reino Aventura Park in Mexico to the Oregon Coast Aquarium and then, in September 1998, from the Oregon Coast Aquarium to a sea pen in Vestmannaeyjar, Iceland, in the same Klettsvik Bay where Little White and Little Gray have struggled to acclimate to the ocean environment [13]. Here, Keiko was housed in an elongated octagon sea pen measuring 100 ft (30.48 m) wide, 250 ft (76.2 m) long, and 30 ft (9.144 m) deep at its maximum [13]. According to those who worked with the animal, including Keiko’s trainer Mark Simmons, the animal seemed to fare well in his adjustment to the same cold weather conditions that have, to date, been problematic for Little White and Little Gray [13]. However, the temperature of his natal waters seemed to be one of the few things about being a wild orca that Keiko adjusted to well. According to Simmons, the managers at the Free Willy/Keiko Foundation expected Keiko to take the initiative toward his own freedom. Perhaps an eliciting call from a relative or the sight of his own kind would spur his orca instincts to drive him away from humans and back to the world he knew as a 3-year-old in the late 1970s. Given what was known in the late 1990s about orcas, one might consider this a plausible hypothesis, but most certainly not a foregone conclusion. Unfortunately, the possibility that Keiko’s reliance on humans (for almost 30 years) might shape his behavior more than instinct was not addressed in his release protocols [13]. As such, no plan was in place for Keiko should he not take the lead in his own release. This left the Free Willy/Keiko Foundation without a strategy for months as plans to have humans teach an orca how to be a wild animal were put into place [60]. Showing disagreement with the idea that long-term captive whales are not capable of innate appreciation and adaptation to the wild, in 2001 Ric O’Barry (of Ric O’Barry’s The Dolphin Project) commented that:

*“At least Keiko is back in home waters, experiencing the natural sounds of the sea and the rhythms of the tide. But he’s still being exploited. The whole plan to ‘train’ Keiko to be wild is ridiculous, a captive idea, and the main problem. They’re putting on a whale rehabilitation show, not letting the whale go wild”* [60].

What people on the ground working with Keiko realized, and what Mr. O’Barry continued to struggle with despite his own issues with releasing the dolphins Buck and Luther in 1994 from his Sugarloaf Dolphin Sanctuary [61], was that cetaceans under long-term human care do not adopt the behaviors of wild whales readily. According to Simmons, the largest impediments to Keiko’s successful integration in the wild were rooted in his relationship with humans [13]. Keiko needed to have an aversion to the physical things humans made (boats, docks, etc.), the sounds humans made, and the humans themselves. Keiko needed to prefer the contact of his own kind rather than that of people [13]. Unfortunately for his rewilding, the whale had 30 years of positive reinforcement paired with everything human. One might argue that Keiko’s poor conditions in Mexico could hardly have been seen as “reinforcing” from an outside human’s perspective, but this was a perspective Keiko did not have. This highlights a concerning philosophy regarding sanctuaries. The assumption is that more natural is better. We do not know if that is a valid assumption for the animals. What is natural to a whale under managed care for decades? Is natural the sound of the tides rolling back and forth, a preference shaped by genes or preexisting sensory biases? Or is natural the sound of *Jump* by Van Halen, a particular anthropogenic sound paired positively to a relationship with a human who is a source of interspecies socialization, stimulation, physical contact, and food? For Keiko, natural may have meant humans.

The plan was never to teach Keiko to be a wild whale by showing him how to hunt and behave like a wild whale. The team in charge intended to offload that responsibility to an ultimately unwilling pod of wild whales that might shoulder that burden [60]. Attempts were made in 2000 and 2001 to integrate Keiko with wild whales. In these initial efforts, both the wild animals and Keiko actively avoided each other [10]. In 2002, Keiko showed more interest, while the wild whales showed more tolerance as the distances between the wild whales and Keiko diminished. Because Keiko was observed logging at various distances from the wild whales (with only one instance of contact recorded), it was assumed that he did not integrate with that group. The one observed example of physical contact was described as such by Simon et al., 2009:

*“There was a splash from the tail of one of the wild whales, which was swimming ventral side up, with his head below Keiko, while he was at the surface. The splash was accompanied by a “startle” reaction from Keiko who swam to the tracking boat, while one of the female/juvenile whales surfaced after him”* [10].

Keiko only spent about a month on his own from 2 to 29 August 2002. While he was in seemingly good body condition upon his reevaluation by humans, it is unknown if he fed during that month. What was certainly true was that he was not in the company of his own kind. Rather, on 30 August he was observed meters from shore, in very shallow water close to Kristianssund, Norway. On 1 September, he followed a small pleasure boat to Skålvikfjorden, where he remained for the rest of his life, engaging large crowds who were touching and swimming with him. During this period, he would initiate contact and swim from one group of people to another. As his time progressed in Skålvikfjorden, the attention from humans became more overt and aggressive, and Keiko’s activity dropped accordingly. From 4 to 9 September, he remained motionless between two skiffs tied to a floating bridge. During this time, people from the area fed him twice. On 6 September, local authorities issued legislation limiting human contact with Keiko. On 8 September, feeding from Free Willy/Keiko Foundation caretakers resumed with boat-led tours of Skålvikfjorden Bay starting the next day to try and increase his movement. Over the next month, a makeshift pen was built, and Keiko mostly had free access to both the entire bay, as well as his pen. Over time, he adopted a pattern in which he would swim alone in the bay and then return himself to his pen. He lived like this, cared for by humans, until his death in December 2003, at the age of 26 [10]. Keiko is instructive to a discussion of sanctuaries, especially in long-term managed animals or animals born under professional care because his behavior after release was completely up to him. I think his example should bring caution to the expectation that cetaceans, even when given unlimited space, will cast off their dependance on humans and be satiated with what we define as a natural existence. Keiko had all the flora and fauna to keep him stimulated and entertained, yet he chose what he knew, humans. Can we expect that cetaceans brought to sanctuaries will be placated by flora and fauna, or will they too seek the attention of humans, the source of food and stimulation for almost all their lives?

Apart from Umah Lumba Rehabilitation, Release, and Retirement Center (i.e., “The Bali Sanctuary”), no active or proposed sanctuaries have released or anticipate releasing animals. However, Keiko is an important datapoint when considering how we should respect learned history in defining what is natural for an animal under long-term managed care in a sanctuary. We are in serious jeopardy of potentially making the naturalistic fallacy regarding cetacean needs in a sanctuary. If we make the argument that these are complex, big-brained animals, then we should not be surprised when we see large aspects of their behavior and preferences driven by learning rather than basic instinct or modal responses [62]. This goes for nonmedical behaviors trained in the course of an animal’s life. We saw with Keiko that just being in a sea pen did not make him fit or healthy [13] and that he needed exercise. Will sanctuaries allow animals to perform high-energy jumps and speed swims to exercise the animals, knowing that these behaviors, despite being natural, represent some of the circus-like “tricks” that have been criticized by activist groups in the past [63]? In her white paper Marino wrote:

*“Many marine parks involve cetacean performances and/or close physical contact with visitors (in petting pools, swim-with sessions, etc.) (Stewart and Marino 2009). Authentic sanctuaries promote respect for autonomy, not requiring any trained behaviors beyond what is necessary for the residents’ health and well-being and allowing only nonintrusive views of the animals (e.g., from a distance or through underwater cameras)”.*

Given that this is a fundamental realignment of the relationship these animals have with humans, I think it is important to ask what exactly those allowed behaviors will be. In addition, what do we do with whales that prefer human contact? Clegg et al. were very clear in their 2018 study that dolphin anticipatory behavior indicates that there is a motivation to participate in trained behaviors, in large part, because of the relationships the animals have built with their human caregivers [46,64,65]. Ultimately the degree to which these interactions are taken away may dictate poorer-quality welfare in sanctuary animals, which is paradoxical to the goals outlined in the GFAS standards. 

## 3. Breeding 

To control breeding with cetaceans, only two classes of options are available: (1) the use of hormonal birth control with medications such as altrenogest (Regu-Mate^®^), medroxyprogesterone acetate (Depo-Provera^®^), and deslorelin (Suprelorin^®^) or (2) sex segregation. There are, however, nuances to both approaches. In Lori Marino’s white paper [24] she stated that,

*“Authentic sanctuaries will work toward a future devoid of the need for sanctuaries for captive animals (by preventing reproduction but not necessarily sexual behavior). Sanctuaries are still part of the captivity spectrum but are distinguished from marine parks by their sole mandate: to restore as much of a natural life to its residents as possible”.*

These two sentences are in direct opposition to each other, as denying reproduction in a species can be one of the most unnatural things one can do. Additionally, this phrasing implies that birth control may be favored over sex segregation, although it is unclear whether she implies same-sex or opposite-sex behavior. Assuming that medical birth control is favored, we will consider that first. 

In bottlenose dolphins, altrenogest is the most commonly used contraceptive. The AZA reproductive management center at the St. Louis Zoo has 117 records for altrenogest use for 53 females. Of these, there are 15 records indicating post-contraception breeding opportunities and 9 records of reversals (post-birth control births). Records exist for 111 deslorelin implant uses in 36 females, and there are 2 records indicating post-contraception breeding opportunities and 2 indicating reversals. There are very few known cases of medroxyprogesterone acetate use in cetaceans [66]. 

Both altrenogest and medroxyprogesterone acetate are synthetic progestin hormones approved for use in horses to regulate the reproductive cycle [67,68,69]. In dolphins, altrenogest is administered orally or through food, and it suppresses ovulation by mimicking the effects of progesterone. By maintaining elevated levels of progesterone-like activity, altrenogest helps prevent estrus in female dolphins. This can be beneficial for managing breeding and reproduction in managed dolphin populations. Altrenogest is also used to synchronize cycles in populations with females and can be a tool for animal managers in artificial insemination when trying to achieve synchronous breeding [70,71,72,73]. Its short-term use in belugas, orcas, bottlenose dolphins, and Pacific white-sided dolphins (*Lagenorhynchus obliquidens* [74]) is somewhat ubiquitous [72,75,76,77]. However, few studies exist on the effects of long-term use of this hormonal birth control in cetaceans, the medication is not 100% effective, and its use in pregnant animals can result in stillbirths and complicated fetotomies [78,79]. The EAAM and EAZA, in their advisory comments on altrenogest [80], regard the medication as safe for short-term use but also highlight that follicular development may be delayed upon cessation of the treatment. The advisory comment also points out that daily treatment is required to suppress estrus and because the medication must be dosed carefully to the weight of the animal (the labeled dose of altrenogest is 0.044 mg/kg), the use of this drug in sanctuaries would mean that the animals would need to be weighed regularly, necessitating a strong husbandry training program in sanctuary animals. Likewise, it is recommended that cetaceans under treatment with altrenogest undergo weekly uterine and ovarian ultrasonographic evaluation, blood work, and reproductive hormone testing (e.g., progesterone, estradiol, etc.) prior to starting the medication and regularly during the treatment due to concerns around uterine inflammation. Regarding long-term use, there is a dearth of information. According to the advisory comments and personal communications with reproductive specialists [81], there are concerns that long-term altrenogest administration could be associated with irregular cycles (longer, shorter, and/or delayed), cyst development, and/or artificially retained corpora lutea. The consensus recommendation from the EAAM and EAZA in order minimize risks of potential side effects of this progestin would be to use it continuously only in sexually mature females (this drug should never be used in pre-reproductive females due to risks of abnormal development) for no longer than the length of a full pregnancy (one year in a dolphin) before allowing the female to cycle at least twice before reestablishing it again. This would make timing very important and necessitate daily close work with the animals, as sonograms and vaginal cytology would be necessary to monitor these cycles [80,81]. It is also essential that animal care personnel wear gloves when working with altrenogest, as it can be absorbed through the skin and cause undesired effects in staff [80]. One area of research that is still needed regarding altrenogest use is how it affects animal behavior. Although this requires systematic study, animals that are on altrenogest are often considered to be more irritable and aggressive, and anecdotally, there have been animal attacks on pool mates during its use [81]. 

The recommended dose for deslorelin is 2 × 4.7 mg or 2 × 9.4 mg, and it comes as an implant. Implants using 4.7 mg last a minimum of 6 months, and 9.4 mg implants last a minimum of 1 year. The use of this method requires female separation for 3 weeks or the use of alternate contraception (Ovaban^®^/Megace^®^) 7 days prior and 8 days after placing the implant to suppress the stimulation phase. There is individual variation in duration of efficacy, so time to reversal can be variable. When inserting deslorelin implants, a successful placement site is beside the pectoral fins, away from fatty tissue, and implants should be removed once they expire to maintain reproductive health [66]. Regarding implant methods of birth control, they require insertion, which always comes with the possibility of infection. They are known to drift in the body and are difficult to monitor. They need to be reinserted prior to when the control period is up, requiring a potentially tight window of intervention. If the animal has reactions to the hormones in the implants—including uterine infections, inflammation, and/or discharge—the drug cannot simply be discontinued. Finding the implant can also be problematic, and removing it will likely create a significant wound. None of the details discussed here can be found in the GFAS standards, with the section on contraception containing only four bullet points assigning the veterinarian the role of determining the least invasive tool for contraception and describing procedures for dealing with already pregnant females arriving to the sanctuary facility in that condition [11], (p. 29). 

Just as there are concerns around the use of birth control, sex segregation as a method of birth control may also present welfare issues. Bottlenose dolphins have a hierarchical promiscuous/fission–fusion mating/social system characterized by fluid, nonrandom associations [82,83,84,85]. While there are periods of a dolphin’s life that are categorized by voluntary sex segregation, this is not a permanent condition [84,85,86], and it is shaped by a number of factors, including ecological [87], environmental, genetic, and cultural factors [88]. Breeding and sexual behavior is important to bottlenose dolphins. Females have an active clitoris that is thought to function as a source of sexual pleasure [89]. Male social organization is thought to revolve around access to females, with increasing male–male aggression associated with larger male alliances (trios as opposed to pairs) exposed to the presence of increasing numbers of females along predictable socio-geographical boundaries [90]. Males are thought to be more aggressive than females with females (at least in Shark Bay, Australia), expressing some degree of tolerance for intersexual male aggression, as evidenced by rake marks [91]. In non-sanctuary facilities, males and females may be housed separately in mixed sex/age groups in which males and females maintain physical access to each other and in mixed sex/age groups in which animals cannot physically contact either other (but can communicate acoustically, visually, and chemically). While studies are starting to address some of these issues using new, innovative methods [7,65,92,93,94], more research is needed to establish how each of these different housing types affects animal welfare. We know that managed animals in mixed male and female groups have more diversity in sexual behavior while showing little difference in aggression rates between all male and mixed male and female groups [95]. While behavioral diversity is increased as a function of number of individuals, mixes of calves and adults produce the most behavioral diversity (however, behavioral diversity is not seemingly affected by group sex composition) [95]. This highlights that calf rearing and even just the presence of juveniles is important to the maintenance of animal welfare in cetacean groups, even in belugas [96]. We also do not know exactly how chemical communication plays a role in sex-segregated groups linked by water flow, as these animals may receive cues that affect behavior [97,98,99]. It is important to note that orcas vary in population dynamics as a function of food specialized ecotypes [100,101], with Biggs/transient orcas having a more fluid social hierarchy than resident orcas, which seems to explain increases in potential male-on-female aggression in Biggs/transient orcas, where the reestablishment of dominance occurs more frequently (along with females needing to protect their calves more from males) [102]. In southern resident killer whales, age and sex do not seem to influence association network structure, meaning that associations were not strongly organized by age or sex but were primarily structured by matrilineal kinship. Younger animals and females are central to social groupings in this population, and synchronous surfacing rates and physical contact rates (measures of behavioral affiliation) showed significant assortment by age and sex [103]. One could interpret these data as supporting mixed sex groupings; however, there are no longer any southern resident killer whales under human care, and more data on animals under managed care would be necessary to see how such populations (especially of mixed ecotype) have adapted their sociosexual association preferences. In belugas, we have good data with animals under managed care that show that males prefer the company of other males, while females tend to prefer their own company, at least in terms of proximity (females may show female–female affiliation in different, yet undescribed ways) [104]. 

Taken as a whole, the cessation of breeding by birth control may carry risks, and we have a lot left to study on long-term sex segregation in managed cetaceans. One thing that is clear is the artificiality of not having young animals in a population and the decrease in behavioral variability inherent in calf-less groups [95,96]. The goal of sea sanctuaries is to end the practice of captivity, and that is the stated reason for the no-breeding policy [24,105], which would entail the end of young animals under managed care in sanctuaries. To deny breeding and calf rearing to the animals would likely be a welfare cost, as these are important elements of these animals’ lives; see [106] for a framework on understanding net positive welfare. In the messaging around their cetacean standards, the GFAS-stated position is that animal welfare comes first, with Valerie Taylor, the executive director of GFAS saying:

*“Like all authentic sanctuaries, these ocean havens will be ‘Cetacean Welfare First,’ meaning that the animals’ wellbeing, rather than visitor experience or company profit, will always be the priority in animal care, sanctuary design and overall operations”* [107].

One could understand considering welfare over visitor experiences or profit (although, see Section 4. *Funding Sanctuaries*), but the question is: can a sanctuary be “Cetacean Welfare First” and deny their animals reproduction by prioritizing the end of captivity over animal wellbeing? This is an open question and something worthy of serious consideration, especially as one considers decreasing the actual welfare of living animals for a perceived welfare benefit to animals who will never be born. This veers into the ethical question about the welfare of the last cetacean in a sanctuary if the mission of ending cetacean captivity suceeds. 

## 4. Funding Sanctuaries

A cetacean sanctuary is an expensive endeavor. Costs rise dramatically the more isolated the facility is from shore access [13]. According to the Sea Life Trust, caring for two belugas (and their assorted other animals) costs $45,000 USD a month, which includes animal food, daily care, and monitoring from the animal care team, as well as maintenance and essential health checks from specialist vets [108]. Keiko’s care in Iceland amounted to over $3 million USD per year ($300,000 a month) in early 2000s dollars (about $5.5 million in today’s dollars) [60]. Keeping any cetacean under managed care is likely to drain funding resources quickly; however, sea pen-style housing is a particularly expensive method, and it is more involved than just installing a net somewhere. According to 2016 estimates from Animal Welfare Institute personnel, each orca net pen could reach $5 million dollars each, with yearly staffing costs of around $500,000 per pen [109]. Howard Garrett of the Orca Network, in an attempt to rehouse the orca Lolita (Tokitae) into a sea pen in the San Juan Islands (north of Seattle), developed a 17-page plan as part of a lawsuit against the Miami Seaquarium. In his plan, he estimated the movement of the animal alone to cost around $200,000, with yearly costs of $1.5 million in the first year [109].

Each of the sanctuaries listed in Table 1 rely on a donation model for support. The degree of that reliance may vary, with the Sea Life Trust Beluga Whale Sanctuary and National Aquarium likely capable of using resources from their established aquariums and attractions to potentially supplement these endeavors, whereas the Whale Sanctuary Project and Ric O’Barry’s Dolphin Project are likely more reliant on donations. However, each organization has donation links featured prominently on their website, with language directly tying donations to their sanctuary activities. The Whale Sanctuary Project is completely reliant on donations; however, as of 2023, it has only built a visitor’s center. As an organization in 2020, about 32.7% of their operating budget went to executive salaries ($246,666 in total) [22]. This was in a COVID year when they only took in $464,517. According to ProPublica [22], the Whale Sanctuary Project had its best year ever in 2021, with $1,540,241 in donations and $134,000 in salary for its Executive Director and $72,000 in salary for its President and Founder. While the 2021 filings represent a more reasonable trend in the percentage of total operating costs going to executive salaries, the pattern for the Whale Sanctuary Project since its inception in 2016 looks more like the 2020 data than it does the 2021 data, with executive compensation as the largest expenditure at about 31–43% [22]. However, given the current executive funding and the known cost estimates from the Sea Life Beluga Sanctuary and the Free Keiko Foundation, as well as the estimates from the Orca Network and Animal Welfare Institute, the Whale Sanctuary Project, as it is funded now, would likely find it difficult to maintain safe funding levels to support the estimated seven belugas they anticipate caring for (Table 1). This does not include the initial building costs, for which the Whale Sanctuary Project may have some support from companies like Munchkin, in the amount of $1,000,000 [110]. 

Assuming that sanctuaries become the only way cetaceans are held under managed care, and assuming that facilities each keep a donation model, one could conceive of a competition between sanctuaries for the little funding available from the public. This would also cut into funding for wild conservation projects, as a limited number of whale causes would garner enough public attention to fund this way. This funding competition may have serious implications for more ecologically salient cetacean conservation causes. Considering accidental births and potential long lifespans, if the above welfare issues can be sorted, we are still anticipating decades of support for numerous sanctuaries in which no lapse in funding can occur. As we saw, the Whale Sanctuary Project experienced funding issues during COVID-19 and would have been unable to support their animals if they had any. One could say the same is true about zoos and aquariums, however. Because they are not running on a nonprofit donation model and have multiple revenue streams, they may be more resistant to global crisis. However, a key question remains. When a zoo goes out of business, their animals can go to other zoos. What happens when a sanctuary goes out of business, especially if there are not many of them and/or animals cannot be transferred between them? Are the taxpayers expected to cover the costs? Funding challenges for sanctuaries are not unique to cetaceans; dog shelters and horse/primate sanctuaries can also struggle to secure funding to support their facilities [111]. However, housing cetaceans is more logistically fraught with high costs, the necessity for intricate planning, fewer sister facilities with expertise in these animals, and difficulties in relocating animals in emergency situations (especially across international borders like the one between the U.S. and Canada).

## 5. Messaging around Sanctuaries

**Figure 4:**
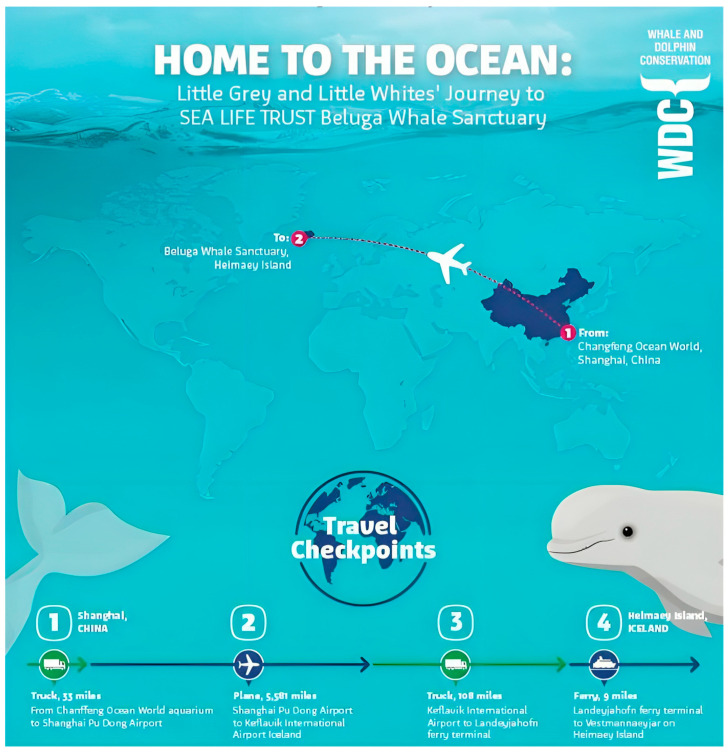
Sea Life Trust partner, Whale and Dolphin Conservation (WDC), Facebook post from 3 September 2018. The Facebook caption read, “Little Grey and Little White are getting ready for their long journey to freedom! They are two female beluga whales that were taken from the wild in Russian waters and are held in an aquarium in Shanghai, in an indoor pool, far from home, on public display to visitors. They are now getting ready to travel 5731 miles to the world’s first beluga whale sanctuary on Heimaey Island in Iceland that we are creating with SEA LIFE Trust. Our aim is to bring the belugas to a safe, natural environment which will support their mental and physical recovery away from their concrete tank and with much greater freedom over the way that they live their own lives!”.

The messaging around sanctuaries often conveys a sense of simplicity, dulling the public into a misguided view that housing animals this way is easy. In some cases, it is also misleading. On 3 September 2018, the Whale and Dolphin Conservation (WDC) published a graphic on their Facebook page promoting the Sea Life Beluga move with a caption beginning with “Little Grey and Little White are getting ready for their long journey to freedom!” (Figure 4). This highlights one of the greatest issues of confusion around sanctuaries in that there is a promise of freedom that is misleading. Sanctuaries are a form of captivity [24], and if freedom is defined as the ability of the animals to go anywhere without barrier, then this is not freedom. Moreover, language like that provides a permission structure for the public to tune out, as justice is now served. This gets back to the problem with the term “sanctuary”. It is a powerful word, one that has the ability to convince a busy and distracted public that refuge and restitution have been achieved for animals that may be perceived as mistreated. As Karen S. Emmerman pointed out in her chapter for Lori Gruen’s *The Ethics of Captivity*:

*“In the public‘s conscience, sighs of relief are common when we hear an animal has made her way to sanctuary. This relief often translates into a sense that the moral work is done with respect to that animal; that the moral universe has righted itself again, and we can turn our attention elsewhere”.*

Therein lies the problem. The word sanctuary can act as a shield to justify the poor treatment of animals. Dr. Emmerman wrote these words concerning the possibility that animals may endure poor treatment, morally justified by the fact that they are promised to a sanctuary at the conclusion of their ordeal, her argument being that placement in a sanctuary is not restitution. However, the moral blindness afforded by the word sanctuary is also a concern after the animals’ sanctuary placement. Many in the public are potentially biased by the word alone and will likely consider the moral imbalance of captivity resolved without considering what is needed for sustaining “good” animal welfare. As such, there may be few, if any, accountability checkpoints on a sanctuary (outside of what is afforded by law) if the public loses its vigilance. In Marino’s white paper she used the term “*restoration*” rather than restitution to again convey that justice is served as an animal is moved into what sanctuary proponents view as a more natural environment (regardless of how the animals perceive it) [24]. Even the use of the word “freedom” by WDC serves this purpose of dulling criticism, as this denies the reality that a sanctuary is just another form of captivity with potential pros and cons worthy of scrutiny. Proper standards under something like GFAS accreditation, therefore, would be crucial to combating this disconnect. However, with guidelines untested by reality, which at times, are seemingly at odds with current best welfare practices, we are left with very little to properly assess the value of this way of housing cetaceans. The word sanctuary cannot be enough.

## 6. The Way Forward for Sanctuaries 

For sanctuaries to be seen as a credible step forward in the welfare of managed cetaceans, more work needs to be performed on determining the best standards for these enclosures through science and experimentation. The word “sanctuary” and its emotional connotations should not deflect criticism of potential welfare deficits or absolve stakeholders from serious self-reflection regarding the potential challenges of this method of housing whales. To ignore these challenges and fail to collect satisfactory experimental data before these animals move to sanctuary enclosures risks not only immediate failure and poor welfare but, coupled with overtly optimistic messaging to the public, also risks stable long-term funding in a donation-based funding model. To achieve this, we must take a true welfare-first approach, rather than an approach that focuses more on the ending of captivity. The GFAS standards highlight how welfare is the first priority, but authors of those standards have argued in the past that ending captivity is their motivation. In an interview with KING 5, Marino, specifically defined the sanctuary as a tool for ending captivity:

*“The sanctuary is an off-ramp for dolphin and whale captivity—and it’s needed because those individual whales who were born into the tanks don’t have the skills to survive”* [105].

Welfare cannot be argued to be the highest priority if it suffers because of a political goal (even an ethical one). Sanctuaries do not have the benefit of decades of zoological operation preceding the development of standards like the AZA or EAZA had. While basic matters seen in zoos for years (like how gates might operate or how acoustic monitoring might work) can be lifted from AZA or EAZA guidelines and put into GFAS standards, where sanctuaries differ from zoological facilities (breeding, space, revenue stream, ethos around human contact, etc.), best guesses currently dictate best practices when it comes to sanctuaries. In truth, we have no idea if the requirements that set a sanctuary apart from a zoo facility equate to best welfare practices or not. That is why studies need to be conducted before standards are developed or, at the very least, concurrent to development. Another focus should be related to gauging the public’s understanding of proposed welfare benefits to determine if long-term supporters of donation-based sanctuary models will likely see their expectations met as NGOs and government partners consider moving forward with cetacean sanctuary experiments.

### Recommendations

We simply cannot assume that just because the ancestors of zoo-housed cetaceans lived and succeeded in the wild that captive-born and long-term captive whales will succeed in sanctuaries. We know that the wild is not ideal for these animals, and moving managed animals closer to a wild environment should not be taken lightly. Regulators and the public should ask the tough questions to spur the research necessary to determine if housing cetaceans in sanctuaries will work. As such, it is imperative that standards be based on the best available data. Where those data are absent, they should be acquired. This should be a noncontroversial statement; however, little is demonstrated in the GFAS standards regarding the knowns and unknowns related to sanctuaries. Even in the AZA standards regarding space for elephants, we see a discussion of what is known and unknown in terms of research on the space and welfare of these animals. No such discussion is found in the GFAS standards, which represents a missed opportunity to push for more science (something that the backers of the GFAS standards at least acknowledge is a focus [112]). For example, we should consider basic habitat preference tests in lagoon facilities that have both pool and sea pen options to determine how animals prioritize and value that kind of space. We need more research on the long-term use of birth control and on the effects of sex segregation of multiple cetacean species (this includes potential chemical transmission and signaling in a sea pen environment) [97]. One could argue that a pool of all male bottlenose dolphins, completely isolated from females, may be different from a welfare standpoint than a sea pen of both males and females in which males are still acoustically and chemically connected to females but physically separated. Acoustically, it is essential to understand how the sounds sanctuary animals produce affect the behavior of wild animals (or vice versa). This may be achieved through both wild and managed-care playbacks to assess recognition and response in both populations [113,114]. Using similar techniques, responses to anthropogenic noise playbacks can be used to determine just how cetaceans in pools will react to noises they have never heard in the wild and, potentially, help prepare them through acoustic habituation [15,114].

In 2020, a new iteration of the five domains model was proposed by Mellor et. al. that included the human dimension [25]. Here, the authors highlighted that the presence of humans can be a positive or negative factor in welfare. Specifically, the authors mentioned that when humans provide preferred foods, structure, enrichment, tactile contact, and/or training reinforcement, humans may be seen as a positive aspect of welfare. Therefore, proponents of sanctuaries should study habitat utilization in pools, lagoons, and existing sea pen facilities either directly or through the literature to try and ascertain how the presence of humans and the reinforcement/structure they provide shapes the need for vast habitats. Furthermore, strong data should be gathered to show that cetaceans under human care will uniformly react positively with increased mental wellbeing in a scenario that a deemphasizes positive relationships with humans in exchange for a relationship with a vast nature, which is fundamentally foreign to long-term captive and captive-born cetaceans. This could involve targeted studies to determine how enriching elements of a natural sea pen would be to a captive-born dolphin (i.e., sounds [114] or objects like sea plants and/or live fish) or a comparative assessment between pools, lagoons, and sea pens to investigate how animals at each of these facility types react to these natural phenomena (i.e., is this something animals in sea pens hardly react to, implying that the novelty will wear off to animals confined in even a vast sea pen). Furthermore, studies should investigate how dolphins who have transferred from pools to sea pens/lagoons and vice versa have fared after their transports. These data are available to be analyzed within existing zoo records. This is essential because there is a body of evidence that suggests that cetaceans in managed care do not use all of their existing environment [115] and that habitat characteristics alone do not predict maximal habitat utilization patterns [116]. Rather, longer dive durations, and use of the bottom third of the habitat is associated with higher enrichment program index values (i.e., more frequent rotations of novel enrichment), as well as socially combining all subgroups at night rather than maintaining the animals in smaller groups [7,116]. Interestingly, behavioral diversity (a measure of positive welfare [117]) is inversely related to depth but positively related to the number of habitats available to the animal and animals that are split into groups and reunited or rotated between subgroups have higher behavioral diversity scores than animals managed in the same group [65]. Both were inversely related to the random scheduling of environmental enrichment [65]. Socialization, on the other hand, is positively related to the frequency of receiving new forms of environmental enrichment but similarly inversely related to enrichment when it is randomly applied [92]. Hence, on some factors, the things that may increase behavioral diversity may decrease habitat utilization, especially regarding social housing [65,116]. Sanctuary proponents should consider what aspects of the Cetacean Welfare Study [7] are applicable to their proposed housing method, specifically the studies above, where the predominate findings are that habitat type or size alone does not dictate quality welfare but rather animal management techniques and timing, which play a much larger role in increasing cetacean behavioral diversity, habitat utilization and socialization scores under human care [7,65,92,116,117].

## 7. Conclusions

Someday, we may need standards for sanctuaries. However, we should not presume that we are in a position today to determine what those standards should be. More research is needed to determine how acres of space, breeding bans, nonprofit donation funding models, and a deemphasis on the human–animal relationship affect animal welfare before we codify these tenets as best welfare practices. Sanctuaries do not have a proven track record of success, and the GFAS standards imply that these prescribes are tantamount to a welfare-first approach. We should be more skeptical of those claims and demand more research into these tenets of the cetacean sanctuary before promoting them as the pinnacle of captive cetacean welfare. Failing to do so risks delegitimizing the sanctuary movement and undermining the safety and care of cetaceans housed in these manners.

## Figures and Tables

**Table 1:** Active and proposed sanctuaries represented in the Global Federation of Animal Sanctuaries (GFAS) standards for cetacean sanctuaries, as well as “The Bali Sanctuary”.

Facility/Sanctuary	Location/Proposed Location	Organization	Species	Current Count	Status
Umah Lumba Rehabilitation, Release and Retirement Center (i.e., “The Bali Sanctuary”)	Banyuwedang Bay, West Bali, Indonesia	Ric O’Barry’s The Dolphin Project *	*Tursiops truncatus*	1 Released (Rambo, presumed alive); 2 Dead (Dewa and Johnny); 1 Missing (Rocky)	Active with no animals
The Beluga Whale Sanctuary	Klettsvik, Iceland	Sea Life Trust (Merlin Entertainment), Whale and Dolphin Conservation	*Delphinapterus leucas*	2 (Little White and Little Grey)	Active with animals in indoor pools
Whale Sanctuary Project	Port Hilford Bay, Nova Scotia, Canada	Whale Sanctuary Project	*Delphinapterus leucas*, *Orcinus orca*?	0 (plans for ~7 Belugas)	Not active, visitor’s center/gift shop built
“National Aquarium Sanctuary”	Unknown as of 2023	National Aquarium	*Tursiops truncatus*	0 (plans for 6, Jade, Spirit, Bayley, Chesapeake, Beau, and Foster)	Not active

* The Dolphin Project was not responsible for GFAS standards but is included here for completeness.

## Data Availability

Not applicable.
